# Value-Based Generic Drug Evaluation Focus on Chinese Real-World Evidence

**DOI:** 10.34172/ijhpm.2023.7970

**Published:** 2023-09-10

**Authors:** Jinmiao Lu, Xiaohua Ying, Zhiping Li

**Affiliations:** ^1^Department of Clinical Pharmacy, National Children’s Medical Center, Children’s Hospital of Fudan University, Shanghai, China; ^2^NHC Key Laboratory of Health Technology Assessment, Department of Health Economics, School of Public Health, Fudan University, Shanghai, China

## Dear Editor,

 Increased use of cheap generics could lead to significant savings in drug spending. In the United States, generics make up 90% of all drugs. More than 95% of China’s 170 000 drugs are generic. Meanwhile, there is a broad class of generic drugs called me-too drugs. More than 60% of the Essential Medicines on the World Health Organization (WHO) list are me-too drugs.^1^ After generics replace branded drugs, we face an impossible trinity. Impossible Trinity refers to drug policy’s difficulty in achieving three objectives (high quality, low price, and adequate supply) simultaneously.^2^ East Asia, Europe, the United States, and the Australian governments have implemented their unique methods for drug quality control and re-evaluation (Figure).

**Figure:**
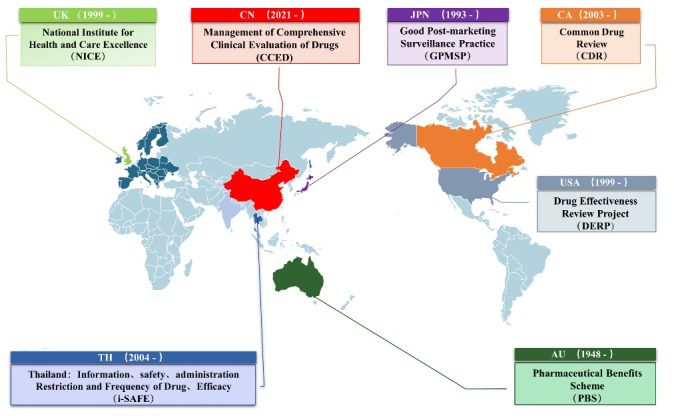
Overview of the Global Post-marketing Medicine Evaluation Program.

 The Chinese government has faced daunting challenges from population aging and rising drug costs. Their policy-makers aim to ensure equitable access to affordable, safe, effective, and sustainable drug supplies. Previously, China’s healthcare authorities used the lowest-priced generic drugs to improve the health of residents. Inevitably, some generic drugs are in short supply or of poor quality. In response, the Chinese Health Commission has strengthened its drug evaluation orientation, shifting from price-based to value-based. Subsequently, the guidelines for the management of Clinical Evaluation of drugs were published in 2021, which is the research focus of drug screening in the Healthy China Initiative from 2021 to 2030.^3^ The results of the evaluation should be applied to the selection and dynamic adjustment of the national essential drug list to control unreasonable drug expenditure.

 China’s drug value evaluation focuses on policy and technology assessment, integrating subjective and objective evaluation. The evaluation considers six dimensions: safety, effectiveness, economy, innovation, suitability, and accessibility. Safety indicators include adverse event rates, while efficacy measures prioritize clinical outcomes. Economic indicators consider drug prices, which can vary for generics. However, consensus has not been reached on evaluation methods and indicators for drug innovation, suitability, and accessibility.

 In the past, there have been flaws in the policy on generic drugs. The effectiveness of some generics has yet to be thoroughly validated, leading to uncertain treatment outcomes. Long-term safety issues have been overlooked, posing risks to patients. Drug pricing and reimbursement policies are imbalanced, causing high prices and limited accessibility. Lack of guidance on drug use can result in misuse. Assessing the value of non-patented drugs faces obstacles such as limited research data, resources, and complexities in the market. Some regulatory agencies prioritize innovative drugs, leading to incomplete evaluation criteria. Limited market competition reduces motivation for evaluation.

 It is possible to use real-world data to evaluate generic drugs. In middle-income countries, little data on solutions from randomized trials.^4^ According to the 2022 China Health Statistics Yearbook, with 3275 tertiary hospitals in China, patient drug use data is enormous. Combining hospital data to generate real-world evidence would be more effective and powerful. There are two apparent barriers to medicine evaluation in China. First, the hospital’s data resources must be retrievable, and data collection and centralization are brutal. As a result, the government has established the National Cancer Center, the National Cardiovascular Center, and the National Children’s Medical Center to set up evaluation bases and collect data automatically. Secondly, the multi-criteria decision-making analysis becomes a model solution to unify subjective opinions and objective clinical data in the evaluation process.^5^ The model-oriented drug evaluation can quickly adapt to real-world data. For example, when the price of a drug is adjusted, the model can update the results sustainably and quickly. Model-oriented pricing and compensation of essential national drugs are becoming vital to a national drug management system. As a decision-making tool, drug evaluation aims to form a comprehensive and universal drug evaluation system.

 The evaluation center collects data from public tertiary hospitals, focusing on real-world applications and patient treatment records rather than pharmaceutical companies. The goal is to have comprehensive coverage across all 23 provinces in China, with at least 100 designated hospitals for data collection. Continuous monitoring allows for the assessment of long-term trends and effects of drug usage. The evaluation results are used to update national drug catalogues, ensuring that only evaluated and approved drugs are included, providing reliable treatment options for patients. Additionally, the evaluation data is a basis for healthcare and insurance institutions to update their internal drug catalogues and guidelines, ensuring access to the latest and most effective medications.

 As data integration improves, breaking barriers between hospitals has become the mainstream direction in medicine. Establishing a medicine evaluation system aims to expand the scope and improve data quality. Future drug evaluations will involve stakeholders from governments, medical institutions, and academia to prioritize population needs and ensure standardized, scientific, and consistent evaluations. Assessing generic drugs using real-world hospital data confirms their safety, efficacy, and equivalence to branded pharmaceuticals.

 We have the following suggestions for government policy-makers. First, using real-world data to compare the clinical effects of generic drugs and original medications can provide accurate evidence and inform policy decisions. Second, conducting budget impact analyses can assess the potential cost savings of generic drug substitution, considering factors like drug prices, patient numbers, and treatment outcomes. Lastly, collecting patient satisfaction data can help understand the acceptance, effectiveness, and side effects of generic drugs, allowing for policy adjustments and guidance. These recommendations will help the government evaluate the effectiveness and cost-saving potential of generic drugs and make informed decisions to promote their appropriate use.

## Ethical issues

 Not applicable.

## Competing interests

 Authors declare that they have no competing interests.
